# Photodynamic Therapy and Cardiovascular Diseases

**DOI:** 10.3390/ijms25052974

**Published:** 2024-03-04

**Authors:** Aleksander Oskroba, Dorota Bartusik-Aebisher, Angelika Myśliwiec, Klaudia Dynarowicz, Grzegorz Cieślar, Aleksandra Kawczyk-Krupka, David Aebisher

**Affiliations:** 1 Science Club, Faculty of Medicine, Medical University of Lublin, 20-059 Lublin, Poland; aleksander.jan.oskroba@gmail.com; 2 Department of Biochemistry and General Chemistry, Medical College of The Rzeszów University, 35-959 Rzeszów, Poland; dbartusikaebisher@ur.edu.pl; 3 Center for Innovative Research in Medical and Natural Sciences, Medical College of the University of Rzeszów, 35-310 Rzeszów, Poland; amysliwiec@ur.edu.pl (A.M.); kdynarowicz@ur.edu.pl (K.D.); 4 Department of Internal Medicine, Angiology and Physical Medicine, Center for Laser Diagnostics and Therapy, Medical University of Silesia in Katowice, Batorego 15 St., 41-902 Bytom, Poland; cieslar1@tlen.pl; 5 Department of Photomedicine and Physical Chemistry, Medical College of The Rzeszów University, 35-959 Rzeszów, Poland

**Keywords:** cardiovascular diseases, photodynamic therapy, photosensitizers

## Abstract

Cardiovascular diseases are the third most common cause of death in the world. The most common are heart attacks and stroke. Cardiovascular diseases are a global problem monitored by many centers, including the World Health Organization (WHO). Atherosclerosis is one aspect that significantly influences the development and management of cardiovascular diseases. Photodynamic therapy (PDT) is one of the therapeutic methods used for various types of inflammatory, cancerous and non-cancer diseases. Currently, it is not practiced very often in the field of cardiology. It is most often practiced and tested experimentally under in vitro experimental conditions. In clinical practice, the use of PDT is still rare. The aim of this review was to characterize the effectiveness of PDT in the treatment of cardiovascular diseases. Additionally, the most frequently used photosensitizers in cardiology are summarized.

## 1. Introduction

Cardiovascular diseases (CVDs), which include ischemic heart disease, stroke, heart failure, peripheral arterial disease and others, have been the main cause of morbidity and mortality in the world for years. According to the analysis carried out in the Global Burden of Disease study, CVD caused approximately 17.8 million deaths worldwide in 2017 [1]. One of the most important causes of cardiovascular diseases is atherosclerosis, which is characterized by the development of atherosclerotic plaques by the subendothelial retention of low-density lipoprotein (LDL) particles containing cholesterol in the arterial wall and by inflammatory changes in endothelial cells. This process may remain clinically asymptomatic for months or even many years [2], and the main risk factors include hypercholesterolemia (LDL cholesterol), hypertension, diabetes, smoking and age [3]. Only damage to the integrity of the arterial surface causes platelet activation, humoral coagulation and the formation of a thrombus, which can close the artery at the site of the ruptured plaque or separate as an embolus and close the arterial lumen in the place below the ruptured plaque [4].

In recent years, numerous efforts have been made to identify clinically dangerous plaques. An early diagnosis of these lesions is made possible by various intracoronary imaging techniques, such as intravascular ultrasound (IVUS), optical coherence tomography (OCT) and near infrared spectroscopy (NIRS), but the development of a safe and effective strategy for the treatment of sensitive plaques remains a key issue [1,2,3,4,5]. Currently, routinely used drugs are unable to prevent up to 70% of clinical events [6]. In the setting of acute coronary syndromes, the most commonly used procedure is percutaneous coronary intervention (PCI) with drug-eluting stents (DES), but these techniques have also been associated with in-stent restenosis, which results from intimal hyperplasia or vascular remodeling and may still occur in up to 2–10% of PCI in some subgroups of lesions patients despite the continuous development and optimization of stent technology [7,8].

Photodynamic therapy (PDT), which is already widely used in the treatment of, among others, dermatological and oncological patients [9,10], is a promising alternative among new therapeutic options for atherosclerosis. The aim of this study is to summarize the current state of knowledge on the use of photodynamic therapy in the treatment of cardiovascular diseases and to evaluate both the safety and effectiveness of PDT.

## 2. Characteristics of Cardiovascular Diseases

Cardiovascular diseases are diseases of the heart, arteries and veins. They may be congenital or acquired [11].

The most common diseases of the circulatory system are hypertension, atherosclerosis, lipid disorders (e.g., high cholesterol), ischemic heart disease and heart attack, heart failure, valvular heart disease, venous thromboembolism, venous insufficiency and varicose veins, chronic limb ischemia (usually lower) and atrial fibrillation (as the most common arrhythmia, i.e., heart rhythm disorder). Cardiovascular diseases are dealt with by doctors of several specialties, including cardiologists, angiologists, vascular surgeons and heart surgeons [12].

**Figure 1:**
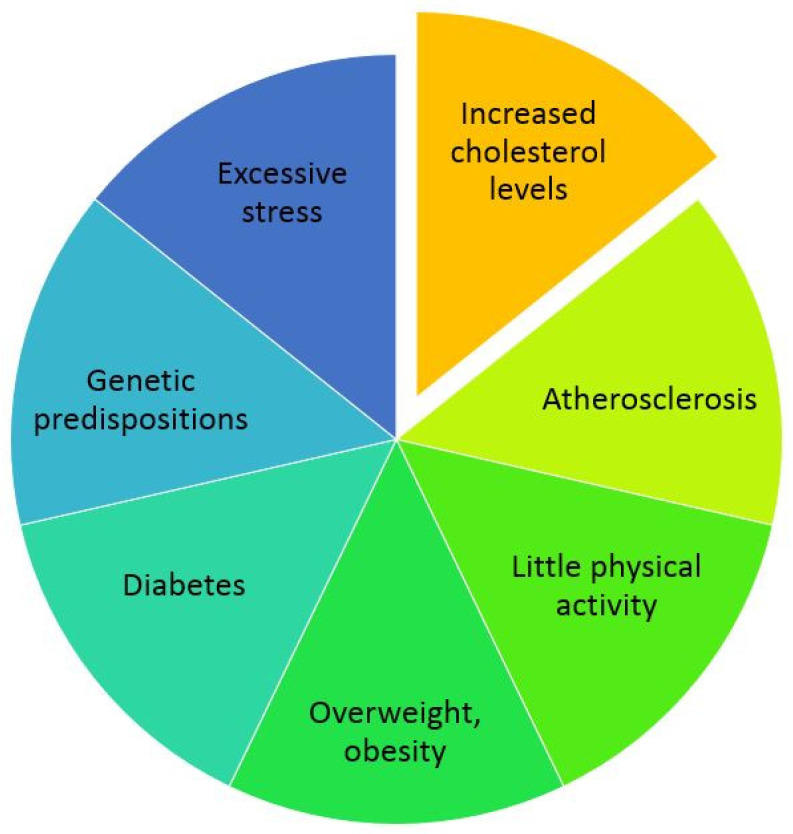
The most common causes of cardiovascular diseases.

Risk factors for cardiovascular diseases include the following: genetic predispositions (early—in men under 55 years of age, in women under 60 years of age—family history of ischemic heart disease or atherosclerosis-related diseases of other arteries), age (men from 45 years of age, women from 55 years of age), gender (higher risk in men than in premenopausal women), little physical activity, increased blood pressure, increased concentration of LDL cholesterol (LDL-C) in the plasma, increased triglyceride (TG) levels, pre-diabetes or diabetes, overweight and obesity (Figure 1) [13]. Other diseases, e.g., of the lungs and kidneys, may also influence the occurrence and course of cardiovascular diseases [14].

The most common type of heart and circulatory system disease is ischemic heart disease [15], the so-called coronary artery disease, which involves limited blood flow to the heart muscle due to narrowing of the coronary arteries resulting from atherosclerosis. The second type of disease is stroke, which involves cutting off the blood supply to the brain; factors of the so-called modifiable risks of stroke [16] include cardiovascular diseases, atherosclerosis of extra-cerebral vessels, including previous myocardial infarction, ischemic heart disease, atherosclerotic narrowing of peripheral arteries, cardiomyopathy and others. 

Currently, there are methods that are aimed at curing atherosclerosis as much as possible as one of the main causes of cardiovascular diseases. In the photodynamic therapy approach, this is a challenge, especially in the ongoing research.

## 3. PDT Mechanism of Action

Photodynamic therapy is one of the therapeutic methods commonly used in the treatment of dermatological, gynecological (breast, endometrial, cervical), urological, gastroenterological and brain cancers [17]. In the treatment of cardiac diseases, there are few references presenting the use of PDT in this type of disease [18]. However, in recent years, several references have appeared describing the effectiveness of PDT in the treatment of atherosclerosis as one of the main causes of cardiac diseases [19]. 

**Figure 2:**
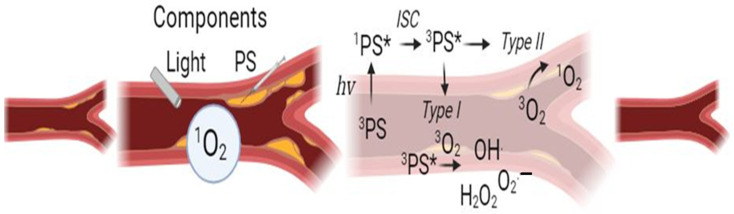
Mechanism of action of photodynamic therapy. When light (hv) is absorbed by PS, the electron transitions from an unexcited low-energy singlet state to a high-energy singlet state. PS can change from the excited state due to photon emission to the ground state due to fluorescence or as a result of internal conversion. An intersystem crossing also takes place in the mechanism, which involves the reversal of the high-energy electron spin, leading to a long-lasting excited triplet state. In the presence of molecular oxygen, superoxide and hydroxyl radicals are formed in type I reactions, i.e., ROS, and in type II reactions, singlet oxygen. S0, photosensitizer ground state (PS); S1, first excited singlet state of PS; S2, second excited singlet state of PS; 3O2, triplet oxygen; 1O2, singlet oxygen.

PDT is a new, promising therapeutic method for atherosclerosis [20]. Its use requires three ingredients: photosensitizers (PS), light with the appropriate wavelength to activate PS and tissue oxygen [21]. The photosensitizer specifically accumulates in the cell and can enter various cellular organelles such as mitochondria, lysosomes, endoplasmic reticulum, Golgi apparatus and plasma membranes [22]. Then, under the influence of light, reactive oxygen species (ROS) are produced, which oxidize cellular components to induce apoptosis [23]. The course of the entire process depends on the type of cell, the photosensitizing agent and its cellular location, as well as the dose of light [24]. Figure 2 shows mechanism of PDT. 

A given photosensitizer can be localized to multiple subcellular locations, thereby causing the simultaneous activation of more than one cell death pathway [25]. Unfortunately, the exact mechanisms that cause the accumulation of photosensitizers in a given tissue are not yet known [26,27]. The photosensitizer can be delivered and accumulated in cells via the low-density lipoprotein (LDL) receptor pathway [28], thus making low-density lipoproteins a promising vehicle for targeted drug delivery [29,30]. Among the factors determining the subcellular localization pattern of a given sensitizer are the physicochemical properties of photosensitizers, such as lipophilicity and charge. Moreover, the way in which the photosensitizer is presented to the cell is important. Porphyrins have been shown to have high affinity for the peripheral benzodiazepine receptor—a protein located in the outer mitochondrial membrane [31,32]. Kessel et al. showed that when a photosensitizer localizes in mitochondria, apoptosis is induced very quickly, unlike photosensitizers located in lysosomes or the cell membrane [33,34]. Studies have observed that after applying PDT to smooth muscle cells of the human aorta, mediators such as Bcl-2 BAX and BAK are released, and the level of mitochondrial cytochrome c (cyt c) and apoptosis-inducing factor (AIF) increases in the cytosol. As apoptosis progresses, there is a cellular redistribution of mitochondrial AIF and the subsequent activation of caspases [35]. These active caspases cleave numerous proteins such as nuclear lamin or poly(ADP-ribose) polymerase (PARP), leading to nuclear breakdown, DNA fragmentation or the inhibition of DNA repair resulting in apoptosis [36]. There is a stabilization and regression of atherosclerotic plaques [37], an inhibition of the development of intimal hyperplasia [38], a selective elimination of macrophages and a reduction in the content of foam cells [39].

### 3.1. Photosensitizers Used in PDT in the Treatment of Cardiovascular Diseases

Many agents have been developed that have photosensitizing properties and can be used in the treatment of atherosclerosis or restenosis (Table 1).

#### 3.1.1. Hematoporphyrin

One of the first agents synthesized, which we now refer to as first-generation photosensitizers, are porphyrin macrocyclics based on hematoporphyrins. In 1983, JR Spears et al. demonstrated the selective fluorescence of aortic atherosclerotic plaques in rabbits and Erythrocebus patas monkeys 48 h after injection of a hematoporphyrin derivative (HPD), with a simultaneous lack of fluorescence in aortic sections free from plaques [40], and in 1986, the selective accumulation of the hematoporphyrin derivative was demonstrated both in vitro in atherosclerotic plaques of patients and in vivo in rabbits with arterial lesions induced by a high-cholesterol diet, catheters or balloon injury [41]. Also regarding the prevention of restenosis, research has been conducted, and it has been observed that PDT using HPD resulted in the significant inhibition of intimal hyperplasia in rabbits with balloon endothelial injury of the common iliac artery [42]. Unfortunately, the main disadvantages of using hematoporphyrin derivatives in clinical settings are cutaneous photosensitivity [73,74] and the insufficient penetration of 630 nm light through endoluminal blood [75]. Therefore, other photosensitizers have also been developed and tested.

#### 3.1.2. Photofrin^®^

This photosensitizer is a proprietary combination of monomers, dimers and oligomers derived from chemical manipulation of hematoporphyrin. The drug has approved indications for the treatment of advanced-stage esophageal cancer, bladder cancer, advanced non-small cell lung cancer and early-stage lung cancer [43] and is also approved by the FDA for use in early and late endobronchial lesions, as well as in Barrett’s esophagus and obstructive lesions of the esophagus, and additional applications include the treatment of bladder cancer [44]. Porfimer sodium has been tested in many preclinical studies both in vitro and in animal models. Studies of the effects of PDT on cultured human smooth muscle cells from human non-atherosclerotic arteries have shown dynamic cellular and cytoskeletal changes in response to irradiation [45], and even the application of the photosensitizer alone without photoactivation resulted in significantly reduced proliferative activity of smooth muscle cells derived from atherosclerotic lesions relative to smooth muscle cells from healthy arteries [46]. PDT studies using Photofrin^®^ (Pinnacle Biologics Inc, Bannockburn, IL, USA) on rabbits with balloon injury showed the inhibition of intimal hyperplasia [76], and a cytotoxic effect on intimal hyperplasia was also observed [77]. In a long-term evaluation of PDT using Photofrin^®^, it was noted that the reduction in intimal hyperplasia in pigs was maintained over a three-to-six-month follow-up period [78]. Various lasers were compared in terms of their effectiveness for photoactivation, e.g., the comparison of the YAG-OPO laser with an argon-color laser. YAG-OPO turned out to be more effective in increasing the diameter of the vessel lumen [79]. YN Hsiang et al. attempted to establish a dose–effect relationship and determined a light dose of 120 J/cm^2^ and a Photofrin^®^ dose of 2.5 mg/kg as optimal for the ablation of atherosclerotic lesions in minipigs while avoiding extensive damage [80,81]. Between December 1999 and February 2000, the first clinical trials of PDT were performed in five patients to prevent restenosis after a coronary stent placement. Porfimer sodium at a dose of 5 mg was administered to the stenting sites in the coronary arteries via a local balloon catheter, and the YAG-OPO laser was tuned to an irradiation of 150 mW/cm^2^. Then, ten minutes after local administration of the photosensitizer, a laser catheter was inserted into the stent lesion. The distal, middle and proximal parts of the stent were irradiated with a pulsed YAG-OPO laser with a total power of 30 J/c. In the period 18–22 months after PDT, no serious adverse events such as death, myocardial infarction or coronary intervention were observed. Importantly, no patient developed photodermatosis, constipation, fever, pleural effusion or anemia. During coronary angiography, performed at the beginning of treatment, after the procedure and after 6 months of follow-up, no restenosis (50%) was observed in any of the stent implantation sites. The stent diameter stenosis was 19.16 +/− 8.20%, and the late lumen loss was on average 0.38 ± 0.18 mm. IVUS analysis revealed intimal hyperplasia with a thickness of 0.3 mm. This study, despite limitations such as a small number of patients or lack of randomization, showed the extremely high effectiveness and safety of PDT using Porfimer sodium in preventing restenosis in the stent. The authors indicated the need to conduct a randomized comparative study of the prevention of restenosis in a stent with and without PDT [82]. 

#### 3.1.3. Verteporfin

Verteporfin, also called benzoporphyrin-derivative monoacid ring A (BPD-MA), is a strong second-generation photosensitizer. Both in vitro and in vivo studies have shown that BPD-MA binds to endogenous low-density lipoproteins and induces apoptosis upon light activation by increasing the levels of mitochondrial cytochrome c and apoptosis-inducing factors [47]. M. Jain et al. observed that after the arterial perfusion of liposomal verteporfin (Visudyne^®^) in the atherosclerotic aorta isolated from mice, followed by PDT, there was significant apoptosis of atherosclerotic plaque macrophages and a weakening of the vascular function of smooth muscle cells [48]. A study by B.A. Allison et al. proved that BPD is taken up by the atherosclerotic plaque in rabbits, although there is no preferential binding of BPD to any particular class of lipoproteins in vivo, and BPD is rather divided according to the plasma concentration of each lipoprotein after release from the liposome. However, the study results suggest that the selective uptake and retention of BPD in atherosclerotic plaque can be achieved and enhanced by combining the photosensitizer with isolated native low-density lipoprotein (LDL) and acetylated LDL (Ac-LDL) carriers. There is a need for further research to better explain the mechanisms of this process [49].

#### 3.1.4. 5-Aminolevulinic Acid (5-ALA)

5-Aminolevulinic acid (5-ALA) is the precursor of protoporphyrin-IX. Studies conducted on both rabbits [50] and pigs [51,52] confirmed that PDT with 5-ALA causes a significant reduction in atherosclerotic plaque and PDT offers a new, promising approach to preventing restenosis after endovascular procedures, as evidenced by a significant reduction in vascular smooth muscle cells (VSMCs) observed 28 days after stenting. A particularly significant reduction in neointimal hyperplasia occurred in the group of rabbits that received PDT before stenting [53]. Thanks to the use of a cylindrical light diffuser, it was possible to achieve a significant reduction in atherosclerotic plaque without damaging the medial wall of the artery [54].

#### 3.1.5. Phthalocyanine Derivatives

Phthalocyanine derivatives represent a class of tetraazaisoindole pigments and are important phototherapeutics due to their desirable optical properties and structural versatility. Studies have shown that the water-soluble phthalocyanine dye is characterized by preferential accumulation in atherosclerotic plaques in rabbits, but the concentration of the dye in atherosclerotic plaques was only 2.6 and 1.7 times higher than in the normal vessel wall after 4 and 24 h, respectively, after the intravenous administration of tetrasulfonate copper phthalocyanines [55]. In a GM study, LaMuraglia et al. observed that PDT using sulfonated chloroaluminophthalocyanine effectively inhibited intimal hyperplasia (IH) in 33 rats that underwent balloon injury [56]. Ortu et al. observed that PDT using chloroaluminium sulfonated phthalocyanine (CASPc) inhibited intimal hyperplasia (IH) in a rat carotid artery balloon injury model. The cross-sectional areas of arterial neointima were measured, and a significant mean decrease in IH was observed in arterial segments irradiated with PDT compared to laser-only controls [57].

#### 3.1.6. Motexafin Lutetium

Motexafin lutetium (Lu-Tex, Antrin Injection; Lu Tex Inc. Seoul, Republic of Korea) is a water-soluble photosensitizer belonging to the texaphyrin family. It selectively accumulates in atherosclerotic plaque, where it can be activated by far-red light, with little skin phototoxicity. In animal models of PDT with motexafin, lutetium significantly reduced at herosclerotic lesions in coronary heart transplant disease [58], significantly reduced the intima/media ratio in early vein graft disease [59] and caused a significant decrease in macrophage numbers and a reduction in atherosclerotic burden without damaging normal vessel walls in a rabbit model of atherosclerosis with balloon injury [60]. Rockson et al. used motexafine lutetium as a photosensitizer in patients with atherosclerotic peripheral arterial insufficiency. Therapy was well tolerated over the entire dose range of motexafine lutetium and light-tested. There were no procedural complications directly attributable to experimental photoangioplasty. Rare side effects were limited to transient paresthesia and minor, transient, self-limited skin eruptions, and no phototoxic symptoms were observed. Researchers suggest that PDT with motexafine lutetium is a promising alternative intervention for the treatment of flow-limiting atherosclerosis [61]. Kereiakes et al. performed phototherapy with Motexafin lutetium (MLu) in patients previously undergoing a percutaneous coronary intervention with a stent placement. Motexafin lutetium was infused into 79 patients [62].

#### 3.1.7. Talaporfin Sodium

Talaporfin sodium is a second-generation photosensitizer used in the treatment of malignant brain tumors. Talaporfin sodium-based PDT induces the occlusion of existing tumor vessels and thus a reduction in tumor blood flow [63,64,83]. In some studies, talaporphyrin sodium has proven to be a probe for visualizing atherosclerosis on the surface of a beating heart in small rabbit coronary arteries [65]. It has also been proven that it accumulates specifically in rabbit atherosclerotic plaques, and when used in PDT, it leads to the destruction of the atherosclerotic plaque skeleton and the lipids accumulated in the plaque [66] and prevents intimal hyperplasia [67]. One of the most important factors determining the success of photodynamic therapy for atherosclerosis is the proper selection of a photosensitizer, which should accumulate in the atherosclerotic plaque and be non-toxic in conditions without light. However, after the activation with light, the photosensitizer should destroy only atherosclerotic cells, without damaging the normal vessel wall. The substance should be safe and must not cause dangerous side effects.

#### 3.1.8. Indocyanine Green 

Indocyanine green (ICG) was initially used to determine cardiac output and liver function and has been used for over 50 years to image retinal blood vessels. It is the only near-infrared dye approved by the U.S. Food and Drug Administration. ICG is amphiphilic, which means that it can interact with both lipophilic and hydrophilic molecules [84]. In 1998, the group of S. Yoney et al. showed that indocyanine green binds intensively to HDL and moderately to LDL in human plasma [85], and in further studies, it was observed that ICG accumulates in inflammatory tissues [86,87]. Verjans et al. demonstrated that ICG is deposited in the atherosclerotic plaques of carotid arteries as well as coronary plaques in diabetic and hypercholesterolemic pigs in vivo. The authors demonstrated the enormous value of indocyanine green-enhanced near-infrared fluorescence (NIRF) imaging for the intravascular imaging of impaired endothelial integrity in human plaques and in vivo in porcine coronary plaques. ICG did not diffusely illuminate all areas of atherosclerosis but rather was deposited adjacent to areas of endothelial impairment with advanced features such as plaque damage or intraplaque hemorrhage [88]. Vinegoni et al. demonstrated that ICG can rapidly target lipid-rich atherosclerosis in cholesterol-fed New Zealand white rabbits, and in in vitro studies using human macrophages, they demonstrated that ICG preferentially targets lipid-laden macrophages, thereby enabling the imaging of sensitive atherosclerotic plaques [68]. However, when assessing lipid-rich inflammatory plaques in rabbits using fully integrated high-speed optical coherence tomography (OCT)/near-infrared fluorescence (NIRF) molecular imaging with indocyanine green (ICG), the authors indicated the need for a further investigation of factors determining ICG uptake by macrophages and binding to lipids. Additionally, imaging limitations have been observed, such as a low penetration depth, an inability to accurately detect the burden of atherosclerotic plaque and positive remodeling and an uneven distribution of ICG, which is probably located more in the intimal areas than in deep tissue parts [69]. ICG has been used as a fluorescent dye for decades and also has great potential as a photosensitizer. When combined with near-infrared light, it causes cytotoxic effects both in vitro and in vivo [89]. E. Engel et al. studied the degradation of indocyanine green (ICG) under the influence of light and the cytotoxicity of ICG degradation products under the influence of light. It has been proven that cells are not killed mainly due to the oxidation of cellular components with singlet oxygen, but due to the toxicity of ICG degradation products [90]. It has also been established that the margin of safety of ICG is narrow, and therefore, we should avoid careless or repeated intraoperative use of ICG [91]. In a 2018 study, Lin et al.’s group conducted research on 15 rats in which a balloon injury (BI) model was used to induce carotid artery intima hyperplasia, and then photodynamic therapy was performed 7 days after the balloon injury, and in one of the groups, the therapy was repeated after 7 days. ICG was administered 1 h before light irradiation with a 780 nm light-emitting diode. It was observed that twice-applied PDT effectively reduces the thickness of arterial walls and the area of intimal hyperplasia and also prevents a reduction in the diameter of the arterial lumen after angioplasty. These findings indicate that repeated PDT with ICG is a novel method for preventing restenosis [70,71,72].

The first challenge in the application of first-generation photosensitizers is their limited penetration and the occurrence of potential side effects, such as photosensitivity. Photofrin seems to be the answer and solution to the limitations of first-generation photosensitizers. However, it also has certain limitations in the therapeutic use of atherosclerosis. Therefore, it is most often tested in in vitro studies using human cells, which bring satisfactory results. A large part of this type of research comes from the years 1990–2000. A small number of literature reports from recent years provoke reflection and constitute a kind of challenge for researchers to develop further research projects in this field. Verteporfin is a photosensitizer more often used in in vivo studies than Photofrin. It is a clinically proven photosensitizer that effectively destroys lipoproteins that contribute to diseases such as ischemic heart disease, stroke and atherosclerosis. The photosensitizer 5-ALA is one of the most popular photosensitizers used in various types of diseases. In the field of cardiovascular diseases, it is used in experimental research in research groups of rabbits and pigs. Like verteporfin, 5-ALA effectively reduces atherosclerotic plaque. In turn, photosensitizers based on phthalocyanine derivatives (as the review shows) poorly accumulate in atherosclerotic plaques. Nevertheless, it is tested in experimental studies on animals, which presents an opportunity and an alternative option to other methods. Motexafin lutetium is one of the few photosensitizers used in clinical trials on patients diagnosed with atherosclerosis. Currently, there are few confirmed in vitro and in vivo studies on the effectiveness of this type of photosensitizer, but from currently published experimental work, it can be concluded that this compound in combination with PDT is a promising alternative option for other forms of treatment. Talaporfin sodium is a photosensitizer with its main use in the treatment of brain tumors. Nevertheless, it is also used in the treatment of atherosclerosis. The cited literature data clearly indicate that it can be included in clinical practice and also in cases of cardiovascular diseases. It is necessary to implement further experimental work that may confirm its effectiveness or, on the contrary, may not confirm the current thesis. The last photosensitizer used in the treatment of cardiovascular diseases is indocyanine green. This type of photosensitizer has been used for decades to image and treat eye diseases (mainly retina). Additionally, there are some experimental data indicating its effectiveness also in the treatment of atherosclerotic lesions. The challenge of using this type of photosensitizer is to understand the course and mechanism of the uptake process by macrophages. Additionally, a hitherto unexplored aspect is the clinical, intraoperative use of ICG and emerging potential side effects, which may prove to be extremely dangerous.

### 3.2. An Antiatherogenic Photodynamic Therapy

Atherosclerotic cardiovascular disease is one of the leading causes of death worldwide [92]. Despite the development of lipid-lowering strategies to help stabilize high-risk atherosclerosis, the risk of the disease remains significant [93]. Detecting inflamed plaque is challenging.

Macrophages play a key role in the development of atherosclerosis, from its initiation to fatal thrombotic rupture [94]. In the arteries, differentiated monocyte-derived macrophages take up lipids, and these foam cells are then presented in the initial stages of atherosclerosis development [95]. Photoactivation, i.e., the process of activating a photosensitizer using laser irradiation, has proven to be a promising therapeutic strategy in the treatment of atherosclerosis, reducing activated macrophages in atherosclerosis and stabilizing atherosclerotic plaques [96]. This process involves using a specific wavelength of light to activate photosensitizers, which leads to the conversion of oxygen into reactive oxygen species (ROS), such as singlet oxygen (^1^O_2_) [97]. ROS generated by light energy eliminate inflammatory cells by inducing apoptosis and support the vascular healing process. However, the unspecific binding of a photosensitizer to vascular structures could potentially lead to unexpected damage to protective barriers, including smooth muscle cells and endothelial cells [98]. Damage to these cells weakens the integrity of the fibrous sheath covering the atheroma, which may lead to thrombosis and a rupture. Therefore, it is necessary to develop a new photoactivated agent with a specific affinity for macrophages.

Macrophage apoptosis is one of the main cellular processes responsible for reducing the inflammatory process in atherosclerosis [99]. It is unclear whether the death of inflammatory cells resulting from photoactivation is actually beneficial and leads to the stabilization of atherosclerotic inflammation [100]. It is worth noting that light energy-induced autophagy activation promotes the degradation of cytoplasmic components in lysosomes, considering that the autophagy mechanism plays a key role in the degradation of phagocytoses of dying cells, and defective autophagy can worsen efferocytosis. Recently, theranostic agents have been developed that target macrophages for the photodynamic and/or photothermal therapy of atherosclerosis. Recent studies show that these agents inhibit the development of atherosclerotic plaques. Apart from inducing apoptosis and reducing platelet burden, these studies have not yet demonstrated phototherapeutic effects on autophagy and efferocytosis in vivo. Inflammatory cells play a key role in all stages of atherosclerosis. Increasing evidence suggests that macrophage apoptosis and the subsequent removal of dead cells play a key role in the treatment of atherosclerosis. The emergence of photoactivation represents a promising alternative for the local treatment of atherosclerosis [96]. Recent studies have identified several new subtypes, e.g., including Mox, M(Hb), Mhem, M4 and HAMac of macrophages, that can be present in atherosclerotic plaques (Table 2) [101].

Several preclinical studies have suggested that effects of PDT using Photofrin^®^ is dependent upon the presence of neutrophils [102]. The pathways leading to the PDT-induced possible mechanism by which macrophages influence of plague is presented in a few steps in Table 3.

## 4. Conclusions

According to extensive evidence based on in vitro and in vivo studies, photodynamic therapy has enormous potential as

(1) A theranostic tool for the identification and regression of sensitive atherosclerotic plaques;
(2) A therapeutic alternative in the prevention and treatment of restenosis.

Despite compelling in vitro evidence supporting the ability of photodynamic therapy to treat de novo or restenotic atherosclerotic lesions, this therapy remains an investigational tool. Several parameters still require further research, such as the optimal photosensitizer concentration, light source and tissue oxygen status. The popularity of PDT testing for non-traditional indications is related to the design of animal models of non-cancer and non-infectious diseases. A diverse cross-section of conditions in cardiology, atherosclerosis, ophthalmology and neuroscience can be treated with PDT. However, before PDT is included in the arsenal of an interventional cardiologist, further research on parameters such as the range of optimal photosensitizer doses and the light source is necessary. The key issue is finding the ideal photosensitizer that will not cause photosensitivity or other dangerous side effects. There is also a need to better understand the mechanisms leading to the death of atherosclerotic cells.

PDT has achieved continued success in oncology. The research is dynamic due to the search for a photosensitizer/drug with the deep-tissue penetration ability of the excitation light for used photosensitizers. Recently, PDT has had a significant impact on the treatment of atherosclerosis. 

## Figures and Tables

**Table 1:** Types of photosensitizers used in PDT in cardiovascular disease.

Photosensitizer	Structure of the Photosensitizer	Absorption Spectrum	Animal Model	Properties	References
Hematoporphyrin	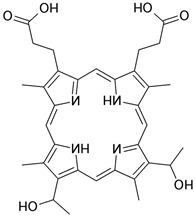	600–650 nm	Rabbits, monkeys	Selective accumulation in atherosclerotic plaques	[40,41,42]
Photofrin^®^	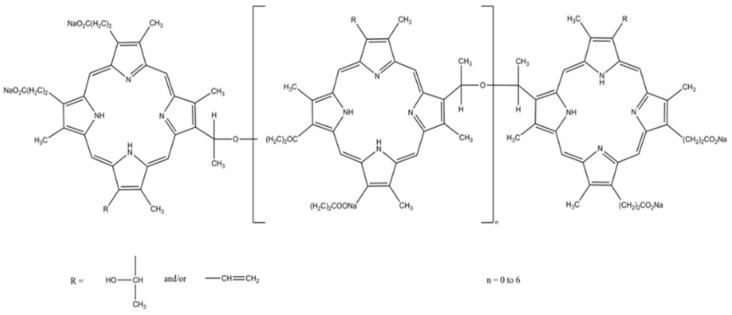	615–635 nm	Rabbits, mini pigs, monkeys	The photosensitizer itself without photoactivation causes significantly reduced proliferative activity of VSMCs	[43,44,45,46]
Verteporfin	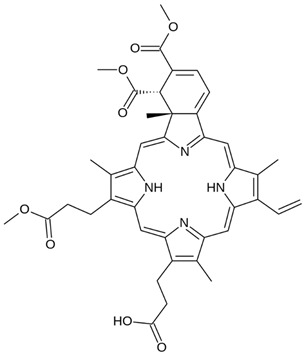	660–700 nm	Mice, rabbits	Induces apoptosis after light activation by increasing the level of mitochondrial cytochrome c and apoptosis-inducing factors, and prevents neointimal hyperplasia	[47,48,49]
5-aminolevulinic acid (5-ALA)	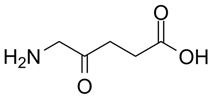	~635 nm	Rabbits, pigs	Significant reduction in VSMC numbers observed 28 days after stenting and reduction in atherosclerotic plaque in animal models	[50,51,52,53,54]
Phthalocyanine	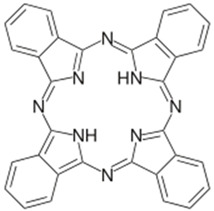	660–680 nm	Rats, rabbits	Effective prevention of IH for up to 6 months	[55,56,57]
Motexafin lutetium	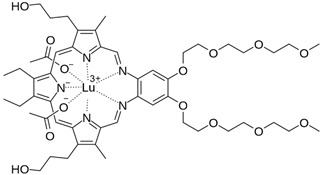	710–750 nm	Rabbits	Significant reduction in atherosclerotic lesions in coronary heart transplant disease, atherosclerotic peripheral arterial insufficiency	[58,59,60,61,62]
Talaporfin sodium	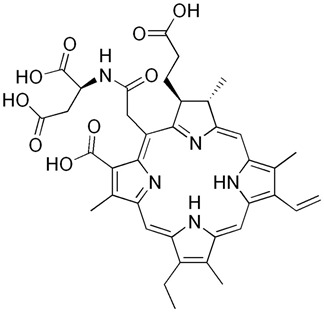		Rabbits	It specifically accumulates in atherosclerotic plaques, prevents neointimal hyperplasia and premature destruction of elastic fiber.	[63,64,65,66,67]
Indocyanine green	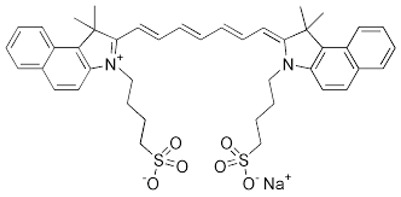	780	Pigs, rabbits, rats	ICG is amphiphilic. It accumulates in inflammatory tissues. It has a small penetration depth.	[68,69,70,71,72]

**Table 2:** Macrophage phenotypes and their effect in atherosclerosis.

Macrophages Phenotype	Role in Atherosclerosis
Mox	Promote heme detoxification Reduce oxidative stress Inhibit foam cell formation
M(Hb)	Scavenge free hemoglobin and prevent its pro-oxidative effects
Mhem	Promote erythrocyte turnover by phagocytosing senescent and damaged erythrocytes, and recycle their iron and heme
M4	Recruit monocytes and neutrophils Degrade extracellular matrix proteins
HAMac	Hemophagocytosis Produce high levels of proinflammatory cytokines and induce apoptosis of smooth muscle cells

**Table 3:** The steps regarding influence of plague by macrophages.

Steps	
A	Macrophages internalize LDL, VLDL and oxidized lipoproteins in the plaque. These processes are known as a macropinocytosi, phagocytosis or scavenger receptor A (SRA), LOX1, SRB1 and CD36 [103].
B	Lipoproteins and their associated lipids are digested in the lysosome. Cholesterol enters the cell membrane, and in the next step, cholesterol is removed from the cell or into the endoplasmic reticulum (ER) membrane. In the next step, is cholesterol is esterified by acyl-coA cholesterol acyltransferase (ACAT) and ultimately stored in this form in cytosolic lipid droplets [104].
C	Lipids can be mobilized for efflux via lipolysis by neutral cholesteryl ester hydrolases (nCEH) or lipophagy, a form of autophagy that results in the delivery of lipid droplets to lysosomes. [105]
D	Cholesterol activates the heterodimeric hepatic X receptor (LXR)/retinoid) or more HDL particles in which the free cholesterol has been esterified and stored in the core of the particle (mature HDL) [106].
E	Cholesterol can induce the formation of cholesterol crystals in the lysosome to activate the NLRP3 inflammasome and can also interfere with ER function (ER stress), which, if prolonged, causes cell death by apoptosis, and the lipid rafts are enriched in sphingomyelin, which forms a complex with free cholesterol [107].
F	As the cholesterol content increases in lipid rafts, proinflammatory Toll-like receptor 4 (TLR4) signaling is promoted, which can also be induced by an oxidized low-density lipoprotein (LDL) via a heterotrimeric complex composed of CD36–TLR4–TLR6. This signaling resultd in the activation of nuclear factor-κB (NF-κB) and the production of proinflammatory cytokines and chemokines [108].

## Data Availability

All data is included in the manuscript.
